# Mutual comparative analysis: a new topography-guided custom ablation protocol referencing subjective refraction to modify corneal topographic data

**DOI:** 10.1186/s40662-020-00201-7

**Published:** 2020-07-07

**Authors:** Kaiwei Cao, Lina Liu, Tao Zhang, Ting Liu, Ji Bai

**Affiliations:** 1 People’s Liberation Army (PLA), No.1441 FengJu Ring Road, Jinniu District, Chengdu, 610081 China; 2 Chongqing vision institute, No. 77 Changjiang 2 Road, Yuzhong District, Chongqing, 400042 China; 3 Department of Ophthalmology, Daping Hospital and the Research Institute of Surgery of the Army Medical University of PLA, No. 10 Changjiangzhi Road, Yuzhong District, Chongqing, 400042 China

**Keywords:** Mutual comparative analysis, Topography-guided custom ablation treatment, Astigmatism, Manifest refraction, Topography

## Abstract

**Background:**

Several planning algorithms have been developed for topography-guided custom ablation treatment (T-CAT), but each has its own deficiencies. The purpose of this study is to demonstrate the potential of a novel mutual comparative analysis (MCA) informed by manifest refraction and corneal topographic data and the patient’s subjective perception in correcting ametropia.

**Methods:**

This retrospective review included patients with significant preoperative differences in the power or axis of astigmatism according to the manifest refraction and corneal topographic data (power > 0.75 D and/or axis > 10°). T-CAT planning was designed using MCA. Follow-ups were conducted for at least 6 months.

**Results:**

Seventy-nine patients (121 eyes) were included. The mean preoperative deviation in the astigmatic power and axis were 0.72 ± 0.43 D and 20.18 ± 23.68°, respectively. The average oculus residual astigmatism (ORA) was 0.81 ± 0.32 D (range: 0.08–1.66 D). Six months postoperatively, the mean spherical equivalent refraction was 0.04 ± 0.42 D, and the mean cylinder was − 0.27 ± 0.24 D. The mean efficacy and safety indices were 1.10 and 1.15, respectively. The uncorrected distance visual acuity in 92% of the eyes was the same or better than the corrected distance visual acuity. The angle of error was ±5° in 61% of eyes and ± 15° in 84% of eyes. Residual astigmatism was ≤0.5 D in 91% of eyes. Optical quality and photopic contrast sensitivity did not change significantly (*p* > 0.05), and the scotopic contrast sensitivity decreased at 3, 6, and 12 cpd (*p* < 0.05). The vertical coma and horizontal coma of the anterior corneal surface significantly decreased postoperatively but increased during follow-up.

**Conclusions:**

The MCA demonstrated safety, efficacy, accuracy, predictability, and stability and can be used as a complementary and feasible method for T-CAT.

## Background

Developed over the course of two decades, topography-guided custom ablation treatment (T-CAT) [1, 2] was initially used for the retreatment of irregular corneas after corneal refractive surgery [3, 4] and the treatment of keratectasia with collagen cross-linking [5].

In 2013, the U.S. Food and Drug Administration (FDA) approved the Contoura vision (CV) method, the WaveLight Excimer Laser System with WaveLight Topolyzer, and a treatment planning software for topography-guided laser-assisted in situ keratomileusis (LASIK) treatment [6].

Several planning algorithms have been developed for the CV system. The FDA algorithm uses manifest refraction as the correction amount during the operation, while a modified FDA algorithm determines the astigmatic axis of the correction based on corneal topographic data. However, the FDA algorithm needs to satisfy the following conditions: (1) for patients with astigmatisms of > 2.00 D, the difference in the astigmatic axis between manifest refraction and topographic data should not exceed 5 degrees, (2) for patients with astigmatisms of < 1.75 D, the difference in the astigmatic axis between the manifest refraction and topographic data should not exceed 10 degrees, and (3) the difference in astigmatic power between manifest refraction and topographic data should not exceed 0.75 D. Due to corneal irregularity, asymmetric astigmatism, or astigmatism in the eye, the power and axis of the astigmatism are sometimes different between manifest refraction and topographic data. The topography-modified refraction (TMR) [7] and Layer Yolked Reduction of Astigmatism (LYRA) protocols [8] have been reported using astigmatic data measured by corneal topography as the correction amount of astigmatism. The LYRA protocol is applied when the manifest refraction and topographic data differ largely in terms of the power and axis of the astigmatism.

Multiple factors can result in a difference in the manifest refraction and topographic astigmatism. Astigmatism correction based on corneal topography alone has deficiencies when the posterior corneal surface or intraocular astigmatism is large [9]. Proposed by Alpins [10], vector planning allows for the combination of manifest refraction and corneal topographic data to correct astigmatism.

Informed by previous methods, we devised a new topography-guided custom ablation protocol to correct refractive errors. Mutual comparative analysis (MCA) combines manifest refraction and corneal topographic data and the patient’s subjective perception. Here, we demonstrated the potential of MCA to correct ametropia.

## Methods

This retrospective study reviewed 121 eyes of 79 patients (29 men) in which myopia and astigmatism were corrected with T-CAT LASIK or T-CAT laser-assisted subepithelial keratectomy (LASEK) at Daping Hospital, Army Medical University of PLA, between August 2016 and August 2018. The preoperative power and/or axis of the astigmatism in these eyes differed between the manifest refraction and corneal topographic data (power > 0.75 D and/or axis > 10 degrees). Follow-up was conducted in each case for at least 6 months. Visual acuity and refraction were recorded, and the objective scattering index (OSI), cutoff for modulation transfer function (MTF), and Strehl ratio (SR) were measured with Optical Quality Analysis System II (OQAS, Visiometrics, Cerdanyola del Vallès, Spain). Contrast sensitivity testing was accomplished using the CSV-1000E (VectorVision, USA). The Pentacam (OCULUS, Germany) is a three-dimensional image scanner and can be used for comparing images at different time points, whereas Topolyzer is a two-dimensional image scanner covering only the anterior surface of the cornea and cannot be used for comparing images at different time points. Since Topolyzer is infrequently used in postoperative examinations, corneal high order aberrations (HOAs) were measured with Pentacam.

The corneas of all patients were preoperatively scanned and processed with Topolyzer Vario and Contoura WaveNet, respectively. Procedures were performed with the Wavelight EX500 Laser (Alcon, US). All surgeries were conducted by the same surgeon (Ji Bai). Surgical planning was completed in the CV system according to the MCA algorithms.

Patients were instructed to wear the bandage soft contact lens for 1 week after receiving LASEK. Anti-infection (levofloxacin eye drops, Santen, Japan; three times daily for 3–4 weeks) and anti-inflammatory (0.5% loteprednol etabonate ophthalmic suspension, Bausch & Lomb, USA; four times daily for 1 week, followed by twice daily for 3 to 4 weeks) treatments were prescribed to each patient in addition to the routine postoperative administration of sodium hyaluronate eye drops (URSAPHARM Arzneimittel GmbH, Germany; four times daily from the first week post-surgery to the third month post-surgery).

All statistical analyses were performed using SPSS 25.0 (IBM, US). Optical quality, contrast sensitivity, and HOAs were compared using paired t-tests. Visual acuity and refraction analyses were performed according to the Standard Graphs for Reporting Refractive Surgery [11]. Standard graphs for reporting outcomes for correcting astigmatism were based on the Alpins Method, and the single angle polar plots were analyzed by AstigMATIC [12].

### The MCA

Corrected visual acuity is a psychophysical response of visual perception and cognition. Through our clinical observations, due to a variety of factors (such as regulation of the ciliary muscle), best-corrected distance visual acuity (BCDVA) is achieved in a refractive range rather than as a fixed value. For example, while astigmatism between − 1.00 diopter cylinder (DC) to − 1.50 DC does not affect patient visual acuity, visual acuity decreases significantly or subjective perception deteriorates if a patient’s astigmatism is beyond the aforementioned range. The astigmatism axis is subject to a similar phenomenon. Although axis adjustment within a certain range has little effect on the visual perception of patients, patients cannot endure axis adjustments beyond the range. Due to regulation, cognitive ability, previous corrective glasses and other factors, the range varies from patient to patient. This feature is the basis of MCA. Based on TMR data (topographic astigmatic power and axis), we set the TMR data as the target and mutually compared TMR data with the manifest refraction.

The MCA is conducted as follows:

1. Optometry and corneal topography examination
2. A difference in the power or axis of astigmatism between manifest refraction and topographic data that exceeds the application conditions of the FDA algorithm
3. TMR data used as the “target,” which can be converted into a frame glasses diopter. Using a phoropter, start with 1/2 or 1/3 TMR or other values, adjust the manifest refractive power and axis of astigmatism so that they approach the “target” while paying attention to the change of the spherical equivalent (SE) and observe the patient’s subjective perception and corrected visual acuity
4. When the power or axis position exceeds a specific value, which varies from patient to patient, corrected visual acuity decreases significantly, or subjective perception deteriorates. This value is used as the correction amount of the surgery
5. If the “target” value (TMR data) is reached and the patient still has no obvious discomfort, then the TMR data are used as the correction amount.

## Results

Of the 121 eyes, 22 and 99 eyes underwent T-CAT LASEK and T-CAT LASIK, respectively. Demographic characteristics are shown in Table 1. Slit-lamp examinations in all patients were normal.

**Table 1:** Demographic characteristics

	Patients	Sex (Male/Female)	Eyes	Age (years)
LASIK	65	23/42	99	27.32 ± 6.83
LASEK	14	6/8	22	24.77 ± 7.20

*LASIK*= laser-assisted in situ keratomileusis, *LASEK*= laser-assisted subepithelial keratectomy

The average deviation of astigmatic power from the manifest to topographic data was 0.72 ± 0.43 D (range: 0.03–1.93 D). The average deviation of the axis was 20.18 ± 23.68 degrees (range: 0 to 89 degrees). The average oculus residual astigmatism (ORA) was 0.81 ± 0.32 D (range: 0.08 to 1.66 D). All astigmatism values were expressed as negative values. Preoperative data on refractive and visual acuity and correction data are shown in Table 2. Among them, 18 eyes (14.88%) tolerated 100% TMR, 30 eyes (24.79%) tolerated 75–100% TMR, 48 eyes (39.67%) tolerated 50–75% TMR, 16 eyes (13.22%) tolerated 25–50% TMR, and 9 eyes (7.44%) tolerated 0–25% TMR.

**Table 2:** Preoperative data on refractive and visual acuity and corrected data

	Mean ± SD	Range
Sphere (M)(D)	-5.29 ± 1.64	-0.75 to -9.00
Cylinder (M)(D)	-0.78 ± 0.68	-5.00 to 0.00
Axis (M)(°)	81.74 ± 69.46	0.00 to 180
CDVA	0.88 ± 0.13	0.10 to 1.0
UDVA	0.16 ± 1.89	0.02 to 1.0
Cylinder (T)(D)	-1.43 ± 0.99	-5.89 to -0.02
Axis (T)(°)	75.77±79.52	0.00 to 180
Sphere (C)(D)	-5.14 ± 1.59	-9.00 to -0.75
Cylinder (C)(D)	-0.99 ± 0.80	-5.25 to 0.00
Axis (C)(°)	75.20 ± 72.39	0.00 to 180.00

*M*= manifest, *T*= topographic, *C*= corrected, *CDVA*= corrected distance visual acuity, *UDVA*= uncorrected distance visual acuity

### General conditions

General conditions such as keratometry, central corneal thickness (CCT), and intraocular pressure (IOP) preoperatively and 6 months postoperatively are summarized in Table 3.

**Table 3:** General condition of preoperatively and postoperative 6 months

	K1 (D)	K2 (D)	CCT (μm)	IOP (mmHg)
Preoperatively	43.05 ± 1.60	44.3 ± 1.58	540.61 ± 25.24	15.21 ± 2.67
Postoperative 6 months	38.76 ± 2.09	39.43 ± 2.17	464.06 ± 30.9	10.71 ± 1.75
p	0.000	0.000	0.000	0.003

*K1*= keratometry flat, *K2*= keratometry steep, *CCT*= central corneal thickness, *IOP*= intraocular pressure

### Visual acuity and refraction

**Fig. 1:**
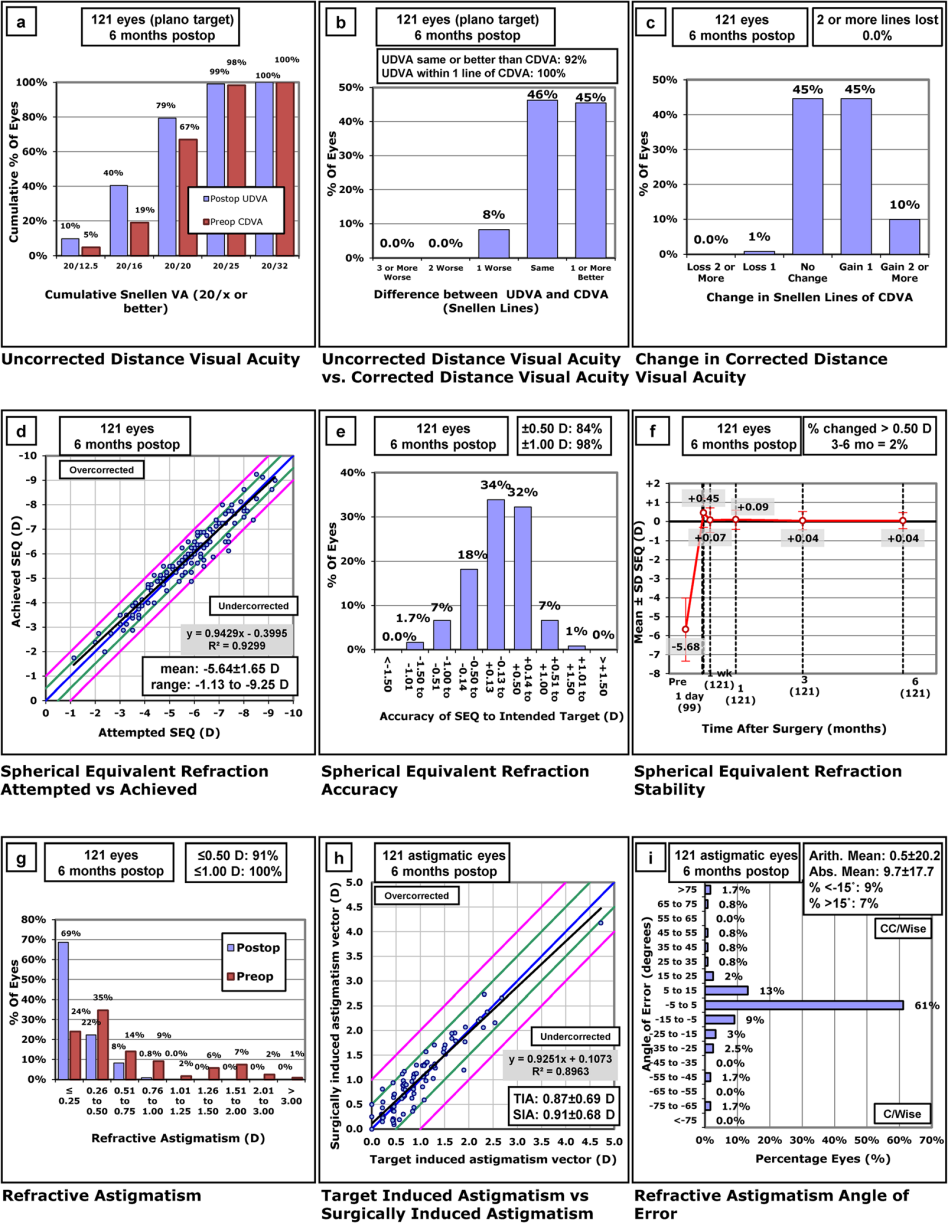
Visual acuity and refraction 6 months post-surgery. **a**: UDVA, **b**: UDVA versus CDVA, **c**: Change in CDVA, **d**: SE Attempted versus Achieved, **e**: SE accuracy, **f**: SE stability, **g**: Refractive astigmatism, **h**: TIA versus SIA, **i**: Refractive astigmatism angle of error Abbreviations: UDVA = uncorrected distance visual acuity; CDVA = corrected distance visual acuity, SE = spherical equivalent; SIA = surgically induced astigmatism; TIA = target-induced astigmatism; Abs = absolute; Arith = arithmetic; C/Wise = clockwise; CC/Wise = counterclockwise

**Fig. 2:**
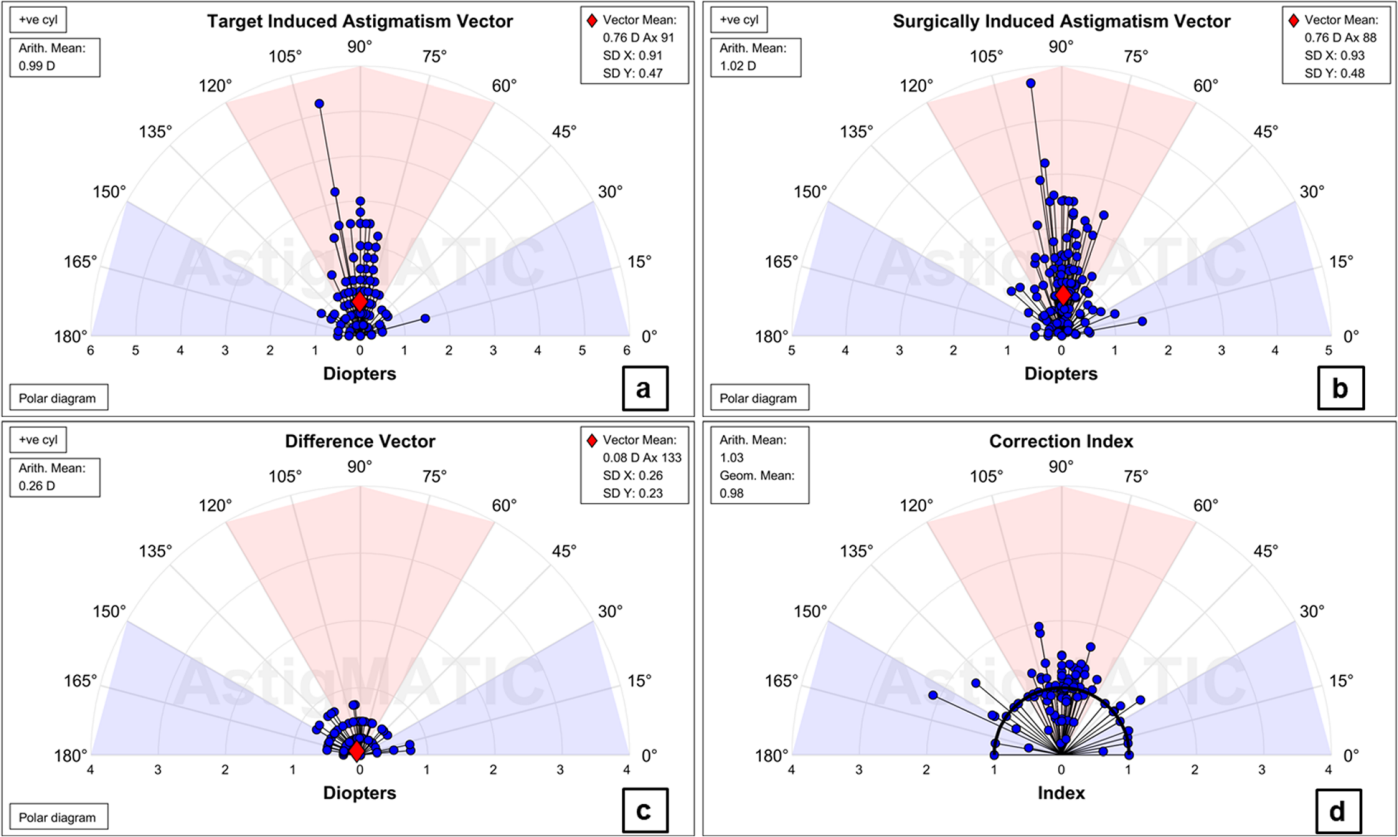
Single-angle polar plots. **a**: Target induced astigmatism vector (TIA), **b**: Surgically induced astigmatism vector (SIA), **c**: Difference vector (DV), **d**: Correction index (CI). The vector means are plotted as a red diamond (calculated in double-angle vector space) and the standard deviations for the X and Y directions are displayed in the call-out box

From all 121 eyes, the uncorrected distance visual acuity (UDVA) was 1.0 or better in 96 (79%) eyes and 0.80 or better in 120 (99%) eyes (Fig. 1a). The mean UDVA was 1.07 ± 0.22 (range, 0.6–2.0). The UDVA was within one line of the CDVA in all eyes (Fig. 1b). The mean efficacy index (postoperative UDVA/preoperative CDVA) of all treatments was 1.10 ± 0.16 (range, 0.75–1.67) after 6 months. The mean postoperative and preoperative CDVAs of all eyes were 1.12 ± 0.21 (range, 0.6–2.0) and 0.98 ± 0.18 (range, 0.6–1.5), respectively. The mean safety index (postoperative CDVA/preoperative CDVA) was 1.15 ± 0.17 (range, 0.75–1.67) in all eyes. One eye (1%) lost one line of CDVA (Fig. 1c). This case underwent T-CAT LASEK and exhibited corneal haze. There was a high correlation between the attempted and achieved SE (Fig. 1d). The mean SE was 0.04 ± 0.42 D (range, − 1.25 – 1.13 D). Of the 121 eyes, 102 (84%) were within ±0.50 D of the intended SE refraction, and 119 (98%) were within ±1.00 D (Fig. 1e). Due to the bandage soft contact lens, refractive data were available for patients who underwent T-CAT LASEK 1-week post-surgery. The mean SE was stable from 3 to 6 months (Fig. 1f). The mean residual astigmatism was − 0.27 ± 0.24 D (range, − 1.00–0.00 D). The residual refractive astigmatism was 0.50 D or less in 110 (91%) eyes and 1.00 D or less in 121 eyes (100%) (Fig. 1g). There was an excellent correlation between surgically induced astigmatism (SIA) and target-induced astigmatism (TIA); the mean SIA was larger than the TIA (Fig. 1h). The mean absolute refractive astigmatism angle of error was 9.7 ± 17.7 degrees, with 101 (83%) eyes having an absolute angle of error of ≤15 degrees (Fig. 1i). Single angle polar plots with vector means for TIA, SIA, difference vector (DV) and correction index (CI) at the 6-month follow-up are shown in Fig. 2.

### Optical quality and contrast sensitivity

The optical quality was measured with OQAS. OSI, MTF, and SR were obtained preoperatively and at 6 months postoperatively (Table 4). No significant difference (*p* > 0.05) was found between the two measurements.

**Table 4:** OQAS results obtained preoperatively and 6 months postop

	Preoperatively (Mean ± SD)	Postoperatively (Mean ± SD)	p^a^
OSI	0.50 ± 0.45	0.50 ± 0.46	0.662
MTF	33.51 ± 9.72	32.79 ± 9.52	0.466
SR	0.22 ± 0.23	0.22 ± 0.19	0.830

^a^ Paired t-test

*OQAS*= Optical Quality Analysis System II, *OSI*= objective scattering index, *MTF*= modulation transfer function, *SR*= Strehl ratio

**Fig. 3:**
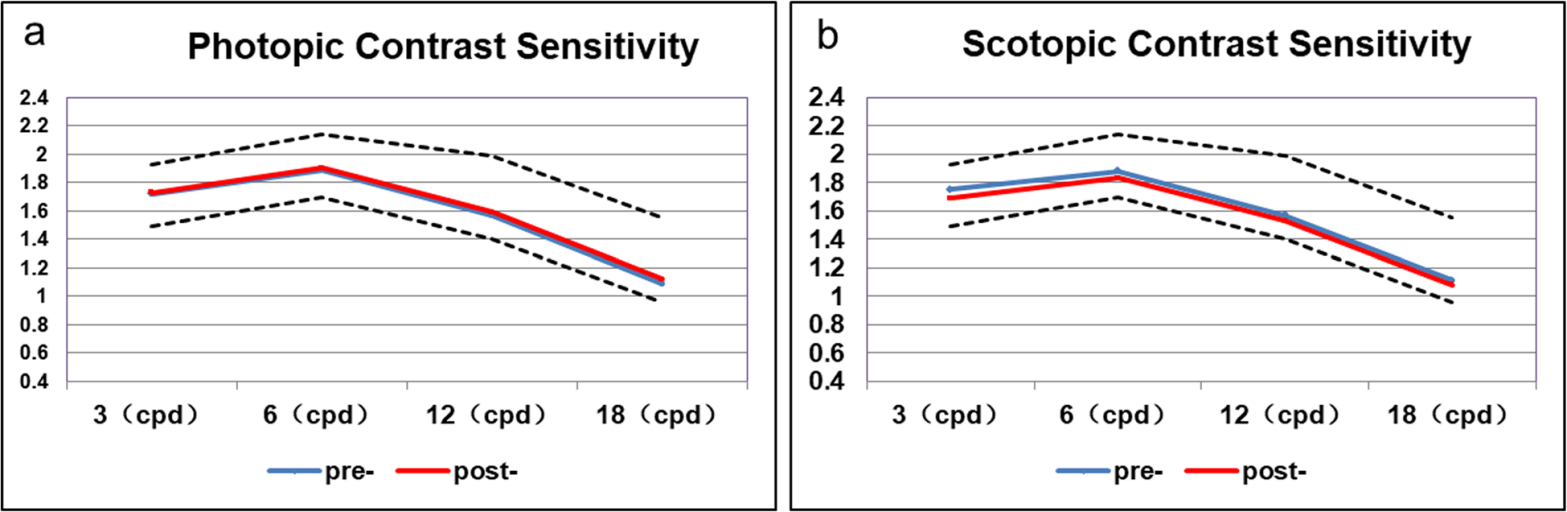
Photopic (**a**) and scotopic (**b**) contrast sensitivities recorded preoperatively and 6 months postop

Photopic and scotopic contrast sensitivity at four spatial frequencies (3–18 cpd) are shown in Fig. 3. Photopic contrast sensitivity did not change at these four spatial frequencies, while the scotopic contrast sensitivity at spatial frequencies of 3, 6 and 12 cpd significantly decreased 6 months postoperatively.

### Zernike analysis

**Fig. 4:**
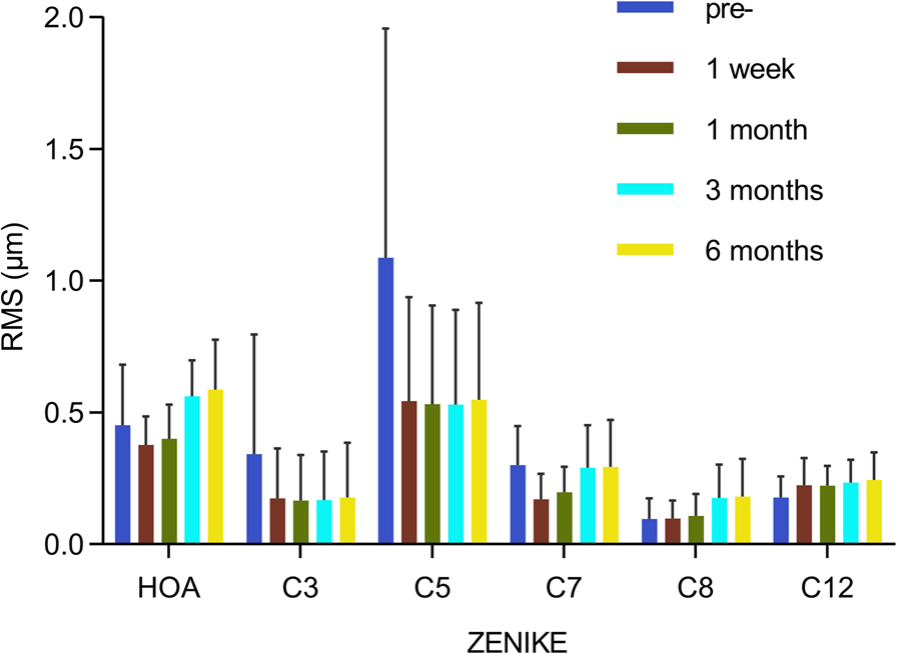
Variation in the magnitudes of the root mean square aberrations at different times. Abbreviations: RMS = root mean square; HOA = higher order aberration; C7 = vertical coma; C8 = horizontal coma; C12 = spherical aberration

Since the transition zone affects aberrations, the Zernike analysis of the anterior corneal surface was performed with a Pentacam at an analysis diameter of 5.50 mm, which was 0.5 mm lesser than the optical zone diameter. The root mean square (RMS) magnitudes of the total high-order aberration (HOA), astigmatism along 45° (C3), astigmatism (C5), vertical coma (C7), horizontal coma (C8), and spherical aberration (C12) were recorded. Variations in these parameters at different times are shown in Fig. 4. The total HOA, C3, C5, and C7 significantly decreased (*p* < 0.05) and C12 significantly increased (*p* < 0.05) 1 week post-surgery relative to the preoperative results (Fig. 3). However, total HOA, C7, and C8 significantly increased (*p* < 0.05) 6 months postoperatively relative to the values obtained 1 week postoperatively. Follow-up examinations revealed that HOAs significantly increased without surgical intervention and that the variations mainly occurred between 1 week and 3 months postoperatively.

### Case example

**Fig. 5:**
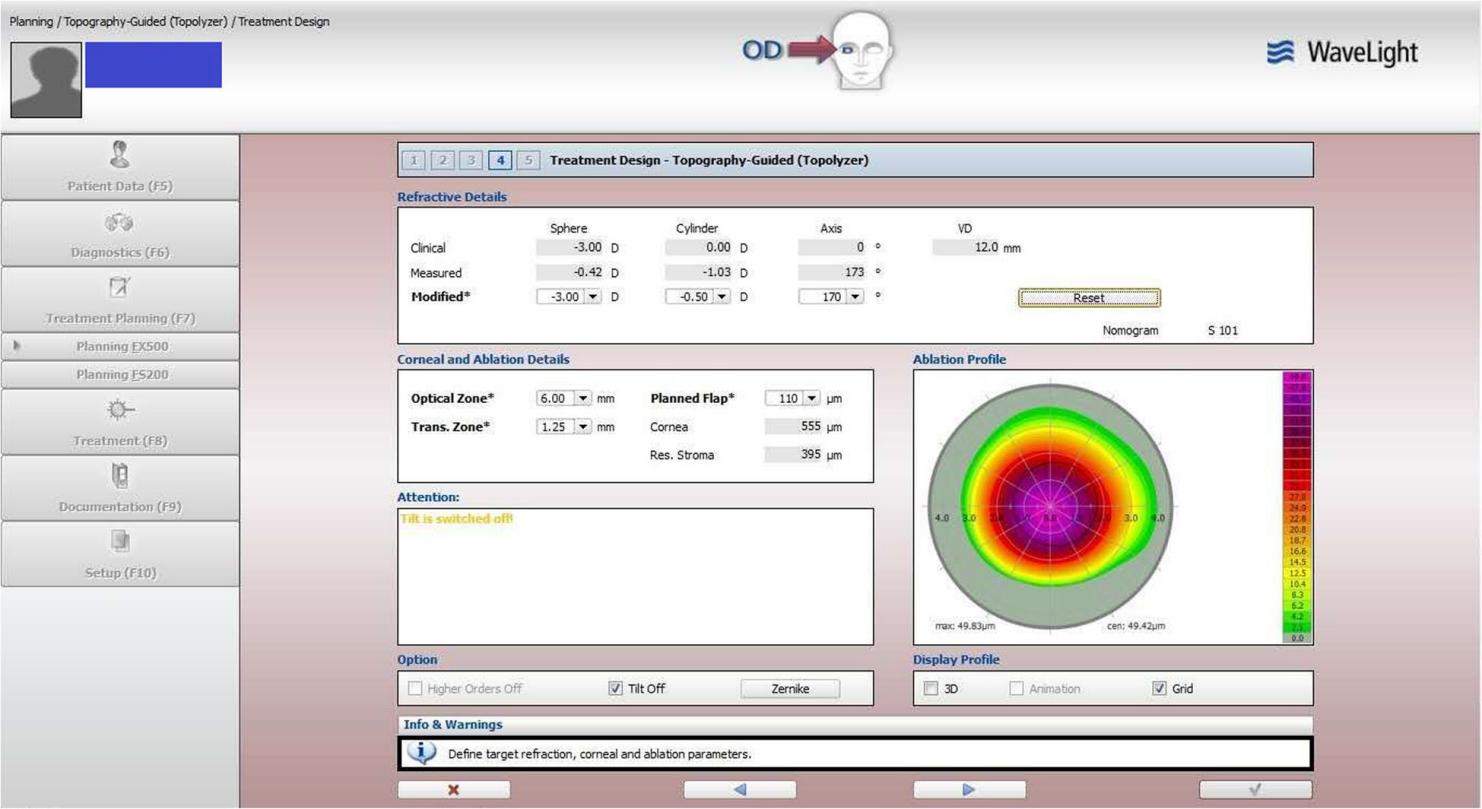
Topography-guided treatment planning of the case example

**Fig. 6:**
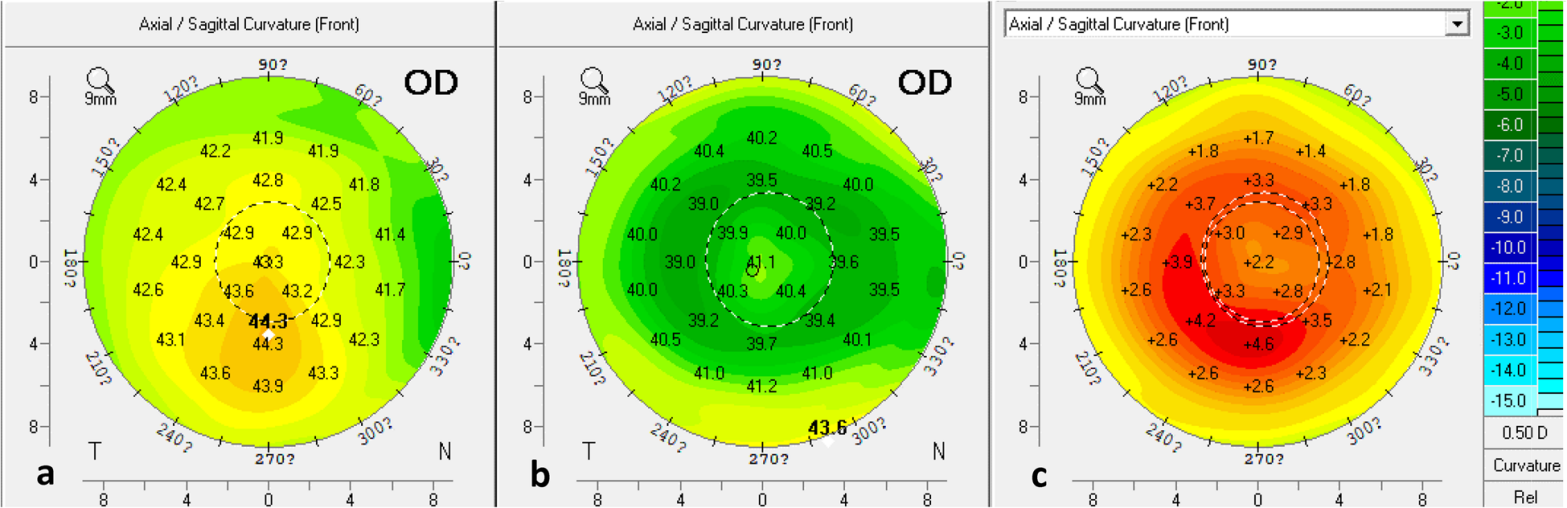
Comparison of corneal anterior surface curvature recorded preoperatively and at 1 week postop for the case example. **a**: corneal anterior surface curvature preoperatively, **b**: corneal anterior surface curvature 1 week postoperatively, **c**: comparison between **a** and **b**.? means degree

A 19-year-old man presented preoperatively with manifest refraction of the right eye of − 3.00 D, 0 D × 0 degree, and the corneal topographic astigmatism measured with the Topolyzer was − 1.03 D × 173 degrees, while the UDVA was 20/63. We set the TMR data as the “target” (in this case, − 1.00 D × 173 degrees), and the optometrist adjusted the manifest refractive power and axis to approach the “target” using the phoropter while paying attention to the change in SE. The CDVA under different refractive values is shown in Table 5. When the astigmatism power exceeded − 0.50 D or astigmatism axis exceeded 170 degrees, the CDVA significantly diminished, and the patient complained of discomfort. After adjusting the refraction back to − 3.00 D, − 0.50 D × 170 degrees, the CDVA did not decrease, and the patient experienced no discomfort. The correction data (− 3.00 D, − 0.50 D × 170 degrees) was then set using the topography-guided treatment planning software (Fig. 5). This case underwent T-CAT LASIK. Manifest refractive results and UDVA were 0.5 D, − 0.25 D × 85 degrees and 20/12.5, respectively, 1 week post-surgery, 0.25 D, − 0.25 D × 84 degrees and 20/16, respectively, 1 month post-surgery, 0.25 D, − 0.25 D × 88 degrees and 20/16, respectively, 3 months post-surgery, and 0.25 D, −0.25 D × 88 degrees and 20/16, respectively, 6 months post-surgery. Corneal anterior surface curvature measured with a Pentacam preoperatively and 1 week postoperatively were compared (Fig. 6).

**Table 5:** Preoperative CDVA obtained under different refractive values in the case example

Refraction	CDVA (Snellen)
−3.00 D	20/40
−2.50 D, −0.25 D × 170°	20/40
−2.75 D, −0.25 D × 170°	20/32
−3.00 D, −0.25 D × 170°	20/32
−3.00 D, −0.50 D × 170°	20/20
−2.75 D, −0.50 D × 170°	20/20
−3.25 D, −0.50 D × 170°	20/32
−3.00 D, −0.75 D × 170°	20/32
−2.75 D, −0.75 D × 170°	20/32
−3.00 D, −0.50 D × 20°	20/40
−3.00 D, −0.50 D × 90°	20/63
−3.00 D, −0.50 D × 140°	20/20
−3.00 D, −0.50 D × 175°	20/32

*CDVA*= corrected distance visual acuity

## Discussion

MCA is a new method for correcting refractive errors calculated using data based on the manifest refraction and corneal topography and the patient’s subjective perception.

Rather than referencing previous studies, the theory of refractive range was evaluated by clinical observation. We searched for theoretical support in previous studies on the visual system, which is composed of the optical and nervous systems; all optical systems exhibit blur to some extent [13], and blur perception is an elemental feature of the human visual system [14]. The field of perception reaches the psychological landscape, and perceptual decisions are based on a posterior probability distribution, which is obtained by combining the likelihood and prior distributions [15]. This combination produced the most mathematically reliable estimate: the maximum likelihood estimate (MLE). The MLE can be obtained from blurred retinal images within a refractive range and the CDVA could thus be achieved within a refractive range rather than as a fixed refractive value. Within the refractive range, there was no significant difference in a patient’s subjective perception.

The CV includes importing treatment data into the laser treatment planning model for T-CAT. The application of topography-guided treatments with this platform have been extensively reported [7, 8].

The FDA algorithm is the most commonly used planning algorithm applied to CV. TMR or LYRA protocols use the astigmatic data measured via corneal topography as the correction for astigmatism. Multiple factors cause manifest refraction and topographic astigmatism to differ: anterior corneal higher-order aberrations, posterior corneal astigmatism, anterior and posterior lenticular astigmatisms, refractive index variability, decentration of the crystalline lens, retinal tilt, and cortical perception [16]. When the posterior corneal surface and intraocular astigmatism are large, astigmatism correction based on corneal topography alone may cause an increase in total eye aberration or corneal astigmatism and tilting of intraocular astigmatism. A recent study [9] also suggested that the TMR and LYRA protocols have drawbacks for correcting ametropia.

Many studies have shown excellent outcomes when planning treatment based solely on subjective manifest refractive astigmatism [9, 17–23]. However, only one study [9] reported differences between manifest astigmatism and topographic astigmatism. The inclusion criteria for most studies did not consider the differences in preoperative power or axis (or both) of the astigmatism between the manifest refraction and the corneal topographic data.

Despite the natural optimal comfort with manifest refraction, this refraction was in a range rather than being a fixed value. We tried to find the correction value closest to the topographic astigmatism within the refractive range with which the patient was comfortable. Combined with the patient’s subjective feelings, MCA maximally corrects the irregularities of the anterior surface of the cornea to achieve a balance between correction of the irregular cornea and the patient’s subjective feelings.

LASEK is applied for correction of low to moderate myopia and thin corneas, and there is uncertainty in how LASEK compares with LASIK in achieving better refractive and visual results [24]. Hence, we put together the results of LASEK and LASIK.

Postoperative results of visual acuity and refraction demonstrated that MCA is safe, effective, accurate, predictable, and stable. The vector analysis of astigmatism (Figs. 1 and 2) showed good results for astigmatic correction. In the present study, one line of vision was gained in 40% of eyes, and two lines were gained in 10%. Additionally, the residual refractive astigmatism (0.50 D or less in 91% eyes and 1.00 D or less in 100%) was consistent with that in two studies in which treatment was based directly on subjective manifest refraction [20, 23]. These findings demonstrate the feasibility of MCA.

The present study found that at 6 months postop, the OQAS results showed no improvement in optical quality, no change in photopic contrast sensitivity, and significant decreases in scotopic contrast sensitivity at spatial frequencies of 3, 6, and 12 cpd. Postoperative corneal HOAs may partly account for the lack of improvement in optical and visual quality.

Repeatability and reproducibility of Zernike measurements on the Pentacam have been reported [25]. Third-order aberrations rather than total aberrations are the main factors affecting contrast sensitivity, and C7 is reportedly negatively correlated with contrast sensitivity and visual quality [26]. In the present study, total HOA and C7 significantly decreased 1 week post-surgery, indicating that the procedures reduced irregularities in the corneas. However, the HOA, C7, and C8 significantly increased during follow-up. Due to the increase in pupil diameter in darkness, scotopic contrast sensitivity may be more susceptible to the increase in corneal wavefront aberration, and thus, account for the postoperative decrease in scotopic contrast sensitivity.

Notably, HOAs increased during follow-up without surgical intervention. This change may be associated with non-uniform proliferation of the corneal epithelium. Two recent studies reported remodeling of the corneal epithelium following corneal refractive surgery [27, 28]. The LYRA protocol also mentions the effect of the epithelium on HOA. Non-uniform proliferation of the corneal epithelium increases the irregularities on the corneal surface and alters the aberrations themselves. Other factors, such as tear film, corneal biomechanics, and intraocular pressure, may also affect the HOA. Optical and visual quality data obtained 1 week postop would have yielded more insights. Nevertheless, further study on the variation in HOAs is needed.

Finally, our study indicated that the selection of the planning algorithm for the CV system could be conducted in the following order: (1) FDA algorithm, (2) MCA can be used as an alternative to current protocols when a patient exceeds the application conditions of the FDA algorithm and the posterior corneal surface or intraocular astigmatism is large.

## Conclusion

MCA was shown to be safe, efficacious, accurate, predictable, and stable. It is a feasible method and can be used as a complementary to T-CAT.

## Data Availability

The datasets used and/or analyzed during the current study are available from the corresponding author on reasonable request.

## Abbreviations

T-CAT: Topography-guided custom ablation treatment
MCA: Mutual comparative analysis
FDA: The U.S. Food and Drug Administration
CV: Contoura vision
LASIK: Laser-assisted in situ keratomileusis
TMR: Topography-modified refraction
LYRA: Layer Yolked Reduction of Astigmatism
LASEK: Laser-assisted subepithelial keratectomy
ORA: Oculus residual astigmatism
OSI: Objective scattering index
MTF: Cutoff for modulation transfer function
SR: Strehl ratio
OQAS: Optical quality analysis system
BCDVA: Best-corrected distance visual acuity
SE: Spherical equivalent
UDVA: Uncorrected distance visual acuity
SIA: Surgically induced astigmatism
TIA: Target-induced astigmatism
DV: Difference vector
CI: Correction index
RMS: Root mean square
C7: Vertical coma
C8: Horizontal coma
C12: Spherical aberration
HOA: High order aberration
MLE: Maximum likelihood estimate
